# Integrated genomics of susceptibility to alkylator-induced leukemia in mice

**DOI:** 10.1186/1471-2164-11-638

**Published:** 2010-11-17

**Authors:** Patrick Cahan, Timothy A Graubert

**Affiliations:** 1 Department of Internal Medicine, Division of Oncology, Stem Cell Biology Section, Washington University, St. Louis, MO, USA

## Abstract

**Background:**

Therapy-related acute myeloid leukemia (t-AML) is a secondary, generally incurable, malignancy attributable to chemotherapy exposure. Although there is a genetic component to t-AML susceptibility in mice, the relevant loci and the mechanism(s) by which they contribute to t-AML are largely unknown. An improved understanding of susceptibility factors and the biological processes in which they act may lead to the development of t-AML prevention strategies.

**Results:**

In this work we applied an integrated genomics strategy in inbred strains of mice to find novel factors that might contribute to susceptibility. We found that the pre-exposure transcriptional state of hematopoietic stem/progenitor cells predicts susceptibility status. More than 900 genes were differentially expressed between susceptible and resistant strains and were highly enriched in the apoptotic program, but it remained unclear which genes, if any, contribute directly to t-AML susceptibility. To address this issue, we integrated gene expression data with genetic information, including single nucleotide polymorphisms (SNPs) and DNA copy number variants (CNVs), to identify genetic networks underlying t-AML susceptibility. The 30 t-AML susceptibility networks we found are robust: they were validated in independent, previously published expression data, and different analytical methods converge on them. Further, the networks are enriched in genes involved in cell cycle and DNA repair (pathways not discovered in traditional differential expression analysis), suggesting that these processes contribute to t-AML susceptibility. Within these networks, the putative regulators (e.g., *Parp2*, *Casp9*, *Polr1b*) are the most likely to have a non-redundant role in the pathogenesis of t-AML. While identifying these networks, we found that current CNVR and SNP-based haplotype maps in mice represented distinct sources of genetic variation contributing to expression variation, implying that mapping studies utilizing either source alone will have reduced sensitivity.

**Conclusion:**

The identification and prioritization of genes and networks not previously implicated in t-AML generates novel hypotheses on the biology and treatment of this disease that will be the focus of future research.

## Background

Therapy-related acute myeloid leukemia (t-AML) is a secondary malignancy attributable to chemotherapy and/or radiation exposure. t-AML comprises 5-20% of adult AML cases and its prevalence is increasing along with the size of the population undergoing chemotherapy [1,2]. While chemotherapy regimen [3] and genetic background [4] contribute to t-AML, the risk factors are not well understood. Strong evidence for genetic predisposition to t-AML is provided by inherited cancer syndromes such as neurofibromatosis, where germline mutations of *NF1 *are associated with increased risk of t-AML in humans and mice [5,6]. Gaining a better understanding of t-AML susceptibility factors is a pressing concern as it may lead to prevention strategies and provide insight into the genesis of *de novo *AML.

One class of chemotherapeutics associated with t-AML is the alkylators (i.e. melphalan, busulfan, thiotepa). The therapeutic effect of alkylator agents is believed to result from the formation of DNA adducts and single and double-strand breaks, which trigger apoptosis or growth arrest [7]. Based on this presumed mechanism of alkylator action, genes involved in DNA repair [8], response to oxidative stress [9], and drug metabolism [10] have been investigated as mediators of t-AML susceptibility in candidate gene studies, with largely inconclusive results. A recent study in our lab investigated the genetic basis of t-AML susceptibility using inbred mice [11]. In this study, eight to twelve individual mice from each of 20 inbred strains were treated with the alkylating agent *N*-nitroso-*N*-ethylurea (ENU), a potent mutagen with a propensity to cause AT:TA transversions and AT:GC transitions [12]. Mice were monitored for the development of AML for up to 16 months post ENU exposure. The incidence of AML varied by strain from 0 to 80% (H^2 ^= 0.10, P-value < 0.001), supporting the hypothesis that there is a strong genetic component in t-AML susceptibility.

We hypothesized that the pre-exposure transcriptional state of hematopoietic stem and progenitor cells, the putative target of leukemogenesis [13], underlies variation in susceptibility to t-AML. A pre-exposure transcriptional basis of susceptibility would be expected if a rapid response is critical in determining a cell's ultimate fate upon mutagen exposure. This hypothesis is consistent with the observation that expression of genes critical to surviving genotoxic stress in yeast does not change after exposure to DNA-damaging agents [14], implying that the necessary factors are already expressed at baseline. A similar situation has been reported in human lymphoblastoid cell lines, in which the pre-exposure transcriptional state of the cell more accurately predicts survival after alkylator treatment than the post-exposure state [15].

**Figure 1:**
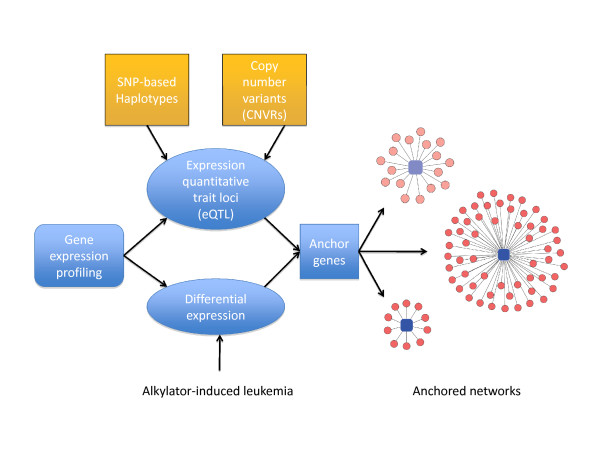
**Data analysis pipeline to identify networks of genes associated with t-AML susceptibility and their putative upstream regulators**. Gene expression profiling was performed on hematopoietic stem/progenitor cells from inbred strains of mice for which t-AML susceptibility has previously been assessed. Expression quantitative trait loci were identified by testing for association between SNP-derived haplotypes or CNVR genotypes (*in cis*) and expression. Genes differentially expressed between t-AML susceptible and resistant strains were identified. Differentially expressed genes that were also associated with eQTL are referred to as anchors and seeded expression network searches.

In this study, we apply an integrated genomics approach [16] to identify and prioritize genetic and transcriptional networks underlying t-AML susceptibility in mice (Figure 1). By linking expression profiles and complex traits to common genomic loci, this method can ameliorate some of the limitations inherent in genetic association and expression profiling studies [17-21]. When combined with network analysis, this methodology has proven useful in elucidating the biological pathways underlying several complex traits [22,23].

## Results

### An integrated genetic map of inbred mouse strains

**Figure 2:**
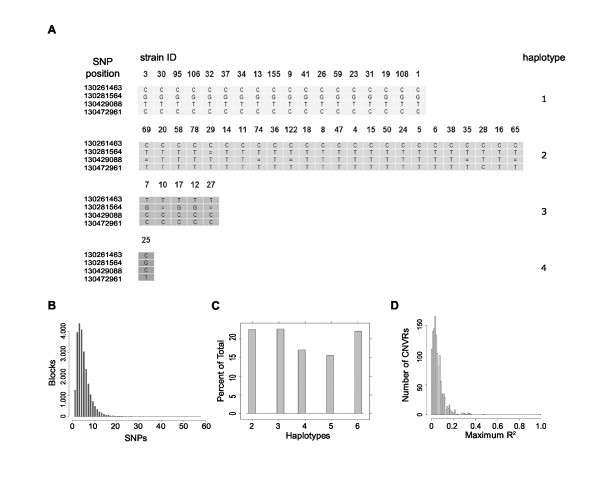
**Mouse Haplotype Map**. **(A) **Typical haplotype block (Block ID 13605, Additional File 1) derived from SNP data http://www.broadinstitute.org/mouse/hapmap/. Rows represent SNPs at the indicated positions on chromosome 4, '=' are untyped. Columns represent 48 classical inbred strains. Mouse Phenome Database strain identifiers are shown for each column. Strain haplotypes are shown on the right. Given the strain haplotypes, it is possible to predict all typed genotypes with at most a single error. The distribution of the number of SNPs **(B) **and haplotypes **(C) **per block. **(D) **SNP-derived haplotype blocks do not tag CNVRs within 250 kb.

Previously, we reported expression quantitative trait loci (eQTLs) in mice using inherited DNA copy number variant regions (CNVRs) as genetic markers [24]. This cis-eQTL map did not explicitly include other sources of genetic variation (i.e., SNPs). To derive a more complete map of cis-eQTLs in this population, we used publicly available SNP data from 48 classical inbred strains to map SNP-based eQTLs http://www.broadinstitute.org/mouse/hapmap/. The SNP resource includes 132,285 SNPs per genome, of which 115,009 we considered informative (as defined in Methods). We used these data to derive haplotype blocks, which are ancestral regions of shared genetic background among strains. Haplotype blocks facilitate trait mapping because they robustly and efficiently represent un-typed genetic variation at a locus. The practical benefit of utilizing haplotype blocks instead of individual SNPs is a reduced number of statistical tests. We used a simple merging algorithm to iteratively join adjacent SNPs into haplotype blocks. This algorithm results in haplotype blocks in which the genotypes of a complete set of SNPs are predictable to a given level of accuracy. We selected a threshold such that for a given block, we can accurately predict the genotype of every SNP in all 48 strains with at most one error (Figure 2A). The 23,884 resulting haplotype blocks are comprised of 1 to 62 SNPs (mean = 4.82, median = 4) (Figure 2B and Additional File 1). The multi-SNP blocks range in length from 18 to 7,618,246 bp (mean = 83,702, median = 43,404). There are 2 to 6 haplotypes per block (mean = 3.92, median = 4) (Figure 2C). Only 21 blocks include haplotypes that are assigned to a single strain. 9,324 blocks have one error, and the remaining 14,560 have zero. Of the 1,262 CNVRs within 250 Kb of a haplotype block boundary, only one CNVR has a genotype that is tagged (R^2 ^> 0.80) by a SNP-based haplotype (Figure 2D). We speculated that the low ability of SNP-derived haplotypes to tag CNVRs was due to the fact that using all 48 classical inbred strains in the haplotype block construction resulted in higher numbers of haplotype labels. Therefore, we also derived a haplotype block map using only the 20 strains from the CNVR study, resulting in a similar inability of SNP-haplotyes to tag CNVRs (data not shown). This suggests that in current databases of genetic variation in the mouse genome, the majority of CNVRs are not captured by SNP resources.

### Global pre-exposure transcriptional state of hematopoietic stem and progenitor cells is associated with t-AML susceptibility

**Figure 3:**
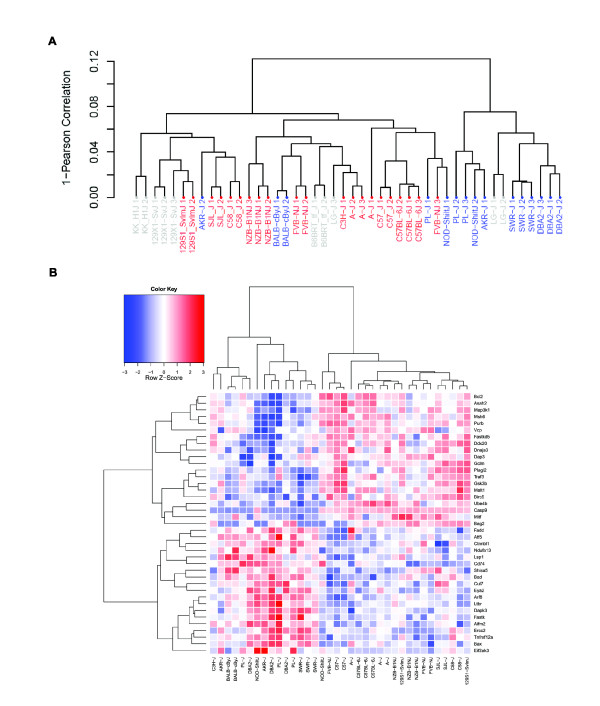
**Gene Expression Profiling of Hematopoietic Stem and Progenitor Cells in t-AML Resistant and Susceptible Strains of Mice**. **(A) **Unsupervised clustering of expression probes that are present in at least 3 strains largely separates t-AML susceptible (blue) from resistant (red) strains. Susceptibility status of some strains is undetermined (grey). **(B) **Differentially expressed genes are enriched in apoptosis-related genes. A heatmap of apoptosis genes differentially expressed between t-AML susceptible and resistant strains is shown.

We performed gene expression profiling (GEP) in c-kit+/lineage- (KL) bone marrow cells (a population enriched in hematopoietic stem/progenitors) from 20 inbred strains listed as Tier 1-4 from the Mouse Phenome Database [25]. Two-to-three biological replicate arrays were analyzed per strain. This gene expression profiling (GEP) data was previously published [24]. Fifteen of the strains were previously assayed for susceptibility to t-AML after exposure to ENU [11]. Unsupervised clustering of gene expression profiles largely separated susceptible from resistant strains (Figure 3A). The probability that the unsupervised clustering of expression profiles predicts susceptibility status by chance is < 0.01 (10,000 permutations, see Methods and Additional File 2). Further, this clustering is not observed in other tissues that are highly unlikely to be involved in leukemogenesis (i.e., the hypothalamus and adipose tissue), nor does it reflect SNP-based strain distances (Additional File 2). Taken together, this supports the notion that the KL clustering of susceptible strains is not due to sequence polymorphism affecting target hybridization [26], but rather reflects tissue-specific differences in transcript abundance between inbred strains [27]. Additionally, this observation suggests that the pre-exposure expression differences of many genes, rather than only a few, segregate the KL cells of t-AML susceptible versus resistant strains.

Next, we asked which genes were differentially expressed between susceptible and resistant strains in KL cells. We identified 917 differentially expressed genes (976 probes) at an FDR threshold of 5% (Additional Files 3 and 4). The differentially expressed genes are enriched in several GO-annotated biological processes (Table 1), including the GO terms 'apoptotic program' and 'nucleotide metabolic process'. The KEGG pathways 'Pyrimidine metabolism' and Colorectal cancer' were also enriched. 'Acute myeloid leukemia' and 'p53 signaling' are biologically plausible pathways that were enriched at least two-fold in the differentially expressed genes, however neither of these pathways passed the FDR < 25% threshold. GO-apoptosis-annotated genes included both cell-intrinsic and extrinsic factors (Figure 3B).

**Table 1:** Functional enrichment of differentially expressed genes

Annotation	Annotation name	Count	P-Value (nominal)	Fold Enrichment	FDR (%)
GO:0008632	apoptotic program	11	9.58E-05	4.71	0.17
GO:0006464	protein modification process	88	1.77E-04	1.46	0.31
GO:0019318	hexose metabolic process	15	0.00107053	2.76	1.88
GO:0005996	monosaccharide metabolic process	15	0.00129867	2.71	2.28
GO:0046907	intracellular transport	43	0.00182315	1.63	3.18
mmu00240	Pyrimidine metabolism	11	0.00325706	2.98	3.99
GO:0031324	negative regulation of cellular metabolic process	25	0.00230736	1.95	4.01
GO:0009117	nucleotide metabolic process	18	0.00246768	2.27	4.29
GO:0009142	nucleoside triphosphate biosynthetic process	9	0.00286827	3.68	4.96
GO:0045934	negative regulation of nucleobase, nucleoside, nucleotide and nucleic acid metabolic process	22	0.00335044	2.00	5.78
GO:0006915	apoptosis	39	0.00643571	1.56	10.81
mmu05210	Colorectal cancer	10	0.00953927	2.74	11.28
GO:0008637	apoptotic mitochondrial changes	5	0.00751505	6.23	12.52
GO:0006396	RNA processing	25	0.00852521	1.76	14.08
GO:0009064	glutamine family amino acid metabolic process	6	0.00927287	4.57	15.22
GO:0015031	protein transport	39	0.01063622	1.51	17.27
GO:0019362	pyridine nucleotide metabolic process	5	0.01370052	5.27	21.69
GO:0008219	cell death	39	0.01444197	1.48	22.73
GO:0016481	negative regulation of transcription	19	0.01461908	1.85	22.98

Count: Number of genes differentially expressed between t-AML-resistant and -susceptible inbred strains of mice with given annotation.

### Integrated cis-eQTL mapping identifies candidate drivers of t-AML susceptibility

Our previous eQTL analysis identified 408 expression traits (391 genes) in KL cells that were associated with 214 CNVRs [24]. We repeated this analysis using the 48-strain haplotype resource to map KL expression traits to SNP-based haplotypes. We considered only cis-eQTL-associated genes, as it has been shown that trans-eQTLs contain a large proportion of false positives [28]. We found 127 associations between expression traits and haplotypes, after selecting the most significant association per trait. In the current study, we used the combined set of SNP- and CNVR-based eQTLs to discover and explore genetically driven modules of co-expressed genes associated with t-AML susceptibility.

There are 45 genes (45 probes) that are both differentially expressed and linked to at least one eQTL. We refer to these genes as anchors throughout the text. 37 are linked to CNVR-eQTLs; the remaining 8 are linked to haplotype-eQTLs. To validate the cis-eQTL associations, we mined publicly available expression data representing hematopoietic stem, progenitor, erythroid and myeloid populations from the BXD recombinant inbred panel [29]. Because this data was generated using the same GEP platform that we used, we were able to ask how our KL population is related to these more purified populations (Additional File 5). As expected, our KL expression profiles cluster most closely with stem and progenitor profiles and are distinct from both erythroid and myeloid lineages. For each anchor gene, we tested the association between BXD genotypes of SNPs within 2 Mb and anchor expression and corrected for multiple testing. We found that 30 of the 45 anchors were significantly associated with at least one SNP within 2 Mb in at least one of the hematopoietic compartments (26 in either Stem or Progenitor), supporting the hypothesis that expression differences of the anchor are caused by locally encoded genetic variation. Of 480 testable eQTLs-transcript associations, 300 (62.5%) were replicated in at least one of the hematopoietic data sets. KL eQTLs may have failed to validate in the other tissues because they are false positives, because the causative genetic variant does not exist in the BXD strains, or due to tissue-specific expression regulation.

### Anchored network analysis identifies t-AML susceptibility expression modules

Next, we hypothesized that expression differences of anchor genes would cause expression differences (in *trans*) on multiple downstream genes (targets). For each anchor, we identified correlated expression profiles (FDR < 1%), resulting in 30 sets of co-expressed genes or modules. The number of targets per module ranged from 3 to 607 (mean = 113, median = 72). We reasoned that true response genes will exhibit association with anchor expression even when the remaining genome is randomly shuffled, as it is in the BXD recombinant inbred cross. For each module, we tested the association between expression of the anchor and each response transcript in each of the BXD hematopoietic populations. We removed target genes from modules that were not associated with anchor gene expression in at least one population (FDR < 25%) (Table 2). We also used a Weighted Gene Co-expression Network Analysis (WGCNA) [30,31], to derive modules of correlated genes independent of linkage to eQTLs. We filtered these modules on the basis of their reproducibility in the BXD dataset and compared the resulting modules with the anchored expression networks. The WGCNA modules are highly similar to the anchored modules in gene content, suggesting that the discovered co-expression structure is robust to different algorithms (data not shown).

**Table 2:** Anchored susceptibility modules

			BXD							
							
Module	Anchor Gene	KL	KLS	KLS-	Gr	Ter	N*	P-value^#^	Top GO	Top KEGG
A_1	LOC634046	460	43	2	23	199	236	2.25	GO:0032940	NE
A_2	scl41743.2_361	402	140	272	207	19	329	1.09	GO:0019219	map04120
A_3	GI_38089999	38	4	1	0	2	7	1.72	NE	NE
A_4	A330106M24Rik	4	3	3	3	1	4	1.82	NE	NE
A_6	Ociad2	132	63	3	16	10	75	1.5	GO:0043666	NE
A_7	GI_46852192-I	97	1	15	1	50	54	1.95	GO:0051329	NE
A_9	Zfp862	106	13	19	1	0	26	2.3	NE	NE
A_12	A630001G21Rik	112	79	50	38	48	104	2.38	GO:0045449	NE
A_14	Aste1	102	7	48	0	1	52	2.67	NE	NE
A_16	Ckap2l	607	4	116	289	4	356	1.28	GO:0006281	map04070
A_17	H2-Ke6	238	12	109	2	81	152	1.93	NE	NE
A_20	Dusp16	91	45	4	43	30	72	2.08	NE	NE
A_21	scl0217069.13_16	58	3	4	21	4	29	2.72	NE	NE
A_22	Atf7ip	39	1	1	9	10	19	2.61	NE	NE
A_23	Snrpn	4	2	2	0	1	3	0.94	NE	NE
A_24	Atp6v0e2	78	4	5	1	0	5	2.91	NE	NE
A_25	Gimap7	30	3	2	0	6	9	2.35	NE	NE
A_26	Pdzk1ip1	79	39	31	0	20	57	2.35	NE	NE
A_27	Polr1b	27	3	10	4	2	12	3.11	NE	NE
A_28	Magohb	65	55	35	24	47	61	0.97	GO:0044242	NE
A_30	Sox13	34	18	25	3	1	29	1.45	GO:0006631	NE
A_32	Ptcd3	18	7	14	5	4	17	2.68	NE	NE
A_33	Casp9	37	1	0	6	1	6	3.22	NE	NE
A_34	Ctsf	223	53	123	8	71	169	2.34	NE	NE
A_36	scl46617.10.1_4	13	4	3	8	5	10	2.5	NE	NE
A_37	Parp2	88	21	21	17	19	42	2.05	NE	NE
A_38	Hdhd3	178	72	2	70	1	102	1.78	NE	NE
A_39	5830417I10Rik	5	1	3	1	1	3	2.07	NE	NE
A_41	Prcp	3	2	1	2	2	3	2.11	NE	NE
A_43	Ggcx	7	6	7	5	3	7	2.53	NE	NE

KL: Number of probes significantly associated with anchored gene expression in kit+/lineage- (KL) cells.

BXD: Number of probes in module significantly associated with anchored gene expression in BXD KLS (Sca+/kit+/lineage-, HSC), KLS- (Sca-/kit+/lineage-, Progenitor), Gr-1+ (Myeloid), or Ter-119+ (Erythroid) cells.

*, Number of probes in module significantly associated with anchored gene expression in at least one BXD data set.

^#^, -Log10(P-value) for association with t-AML susceptibility.

Top GO/KEGG, annotations significantly enriched in each module; NE, not enriched.

**Figure 4:**
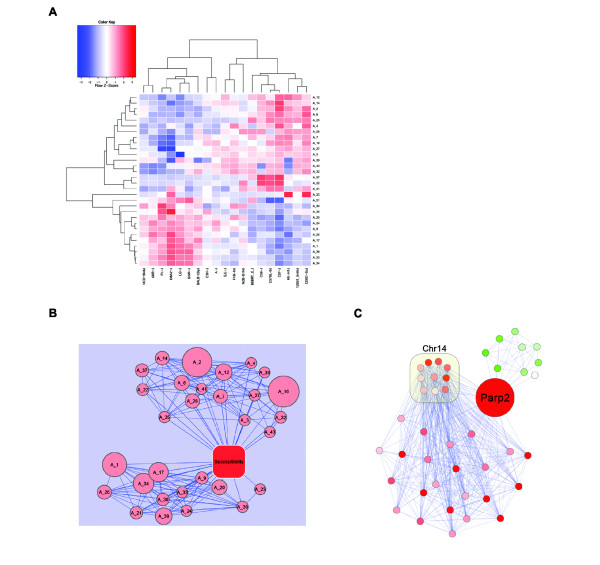
**Anchored Susceptibility Networks**. **(A) **Heatmap of anchored module eigengenes. For clarity, eigengene values were averaged by strain, and each module was row-normalized. Module Eigengenes are either positively (bottom half) or negatively (top half) correlated with susceptibility status. **(B) **Network view of anchored modules. Anchored modules are represented as nodes. Edges between modules represent network eigenegene correlation. Low and negative correlations are not shown for clarity. Edges between the 'Susceptibility' and anchored network nodes represent association between network eigengenes and susceptibility status. Node size indicates the number of response genes in the anchored network. The top super-module corresponds to the top half of the module heatmap displayed in panel A. Likewise, the bottom super-module corresponds to the bottom half of the module heatmap. **(C) **Module A_37, includes 10 genes on chromosome 14 located within 7 Mb of a CNVR. Green nodes represent genes with lower expression in susceptible strains, red nodes represent genes with higher expression in susceptible strains. Correlations among response genes, represented as edges, are only displayed for those relationships where the Pearson correlation > 0.5.

The expression of each anchor gene is, by definition, associated with susceptibility status. However, the strength of the association between the target genes of an anchored module and susceptibility is unknown. To determine these values, we first computed eigengenes from each module [31]. Then, we ranked anchored modules according to differential expression of the module's eigengene and susceptibility status. Using both KEGG and GO annotations, we found that 8 anchored modules were enriched in at least one annotation. We visualized the anchored susceptibility modules as both heatmaps of eigengene values (Figure 4A) and networks (Figure 4B), displaying the correlation between anchored modules and the strength of association between anchored modules and susceptibility status. We also visualized one of the anchored susceptibility networks, focusing on a biologically compelling module (Figure 4C).

## Discussion

There is accumulating evidence that many genetic contributors to complex traits are not protein-coding changes [32,33]. If true, then other classes of genetic events that can affect phenotype must, at some level, impact gene expression (i.e., eQTLs). Hypothesizing that such events contribute to t-AML susceptibility, we took an integrated genomics approach to identify and prioritize candidate transcriptional networks. The first step in this approach was to identify eQTLs in hematopoietic stem and progenitor cells, the likely target of leukemic transformation. Previously, we described a CNVR eQTL map in classical inbred mice [24]. In the current work, we expanded this map to include SNP-based haplotype eQTLs. In deriving the mouse haplotype map, we found surprisingly little correlation between haplotypes and neighboring CNVRs. This is in contrast to human studies, where nearly 75% of common CNVRs are estimated to be in linkage disequilibrium with neighboring SNPs [34]. This suggests that at the currently available resolution and coverage (and genotyping accuracy), mouse haplotypes and CNVRs represent distinct sources of genetic information. We found two-fold more CNVR eQTLs than haplotype-based eQTLs (401 vs. 167). It is tempting to speculate that this difference in eQTL types is because CNVRs have a stronger impact on expression in *cis *and therefore are more likely to be detected as eQTLs. However, the difference could largely be due to the reduced power to detect haplotype eQTLs because of the exacerbated multiple testing problem that comes with performing approximately 20 times more statistical tests. In total, greater than 60% of the eQTLs were reproducible in an independent dataset.

The second step in the integrated approach was to find genes differentially expressed between t-AML susceptible and resistant strains. Because unsupervised clustering using *all *expressed transcripts grouped strains by susceptibility status, we expected to find a large number of genes associated with susceptibility. Greater than 7% of the expressed transcripts are differentially expressed (976/13,496). These genes are enriched in several, independent biological processes, most notably apoptosis. Among the differentially expressed intrinsic apoptosis genes are Caspase 9 (*Casp9*), B-cell leukemia/lymphoma 2 (*Bcl-2*), BCL2-associated agonist of cell death (*Bad*), BCL2-associated X protein (*Bax*), and mutS homolog 6 (*Msh6*). Msh6 is a member of the mutSα DNA mismatch recognition complex that has been shown to mediate apoptosis in certain contexts [35,36]. Notably, the absence of mutSα activity in myeloid progenitors results in the complete loss of *O*6-methylguanine (*O*6MeG)-mediated cytotoxicity [37]. That resistant strains have higher expression of *Msh6 *suggests that upon alkylator exposure, resistant strains may recognize DNA damage and respond appropriately (i.e. undergo apoptosis) whereas the KL cells of susceptible strains may tend to survive, accumulate mutations, and become transformed. In KL cells, almost all susceptible strains have no detectable expression of *Casp9*, an initiator of programmed cell death, suggesting that these cells (low-to-no *Casp9 *expression) are less primed for Casp9-dependent apoptosis. Although knockout of *Casp9 *in mice is embryonic lethal, presumably due to severe brain development defects [38,39], *Casp9 *is not required for apoptosis during normal hematopoiesis [40]. Therefore, inbred strains may exhibit genetically-driven yet tissue-specific differences in apoptosis such that hematopoietic cells of susceptible strains are relatively protected from cell death after exposure to genotoxic stress.

Differential expression and gene enrichment analysis highlighted several biologically plausible pathways that may underlie t-AML susceptibility. However, it remained unclear which pathway members, if any, are causal contributors to the phenotype, as illustrated by the complex expression patterns of the intrinsic apoptosis genes. More broadly, the role and relative importance of each of the 917 differentially expressed genes in susceptibility remained undetermined. We hypothesized that among the 917 differentially expressed genes would be a subset in which expression variation is caused by cis-encoded genetic variation. Further, we posited that these 'anchor' genes cause expression variation of multiple downstream genes, which collectively are associated with t-AML susceptibility (or resistance). The mechanisms by which anchors might act in *trans *are varied. They include altered transcription factor abundance and homeostatic or compensatory forces within and between biological pathways. Regardless of the mechanisms of action, the identification of putative events that influence susceptibility and their linkage to gene networks forms a powerful and practical strategy to both find biological pathways underlying cancer susceptibility and to prioritize candidate mediators. Therefore, as the third step in the integrated genomics approach, we identified networks of genes that are significantly correlated with candidate susceptibility anchors. To validate the networks, we used independent gene expression data from multiple hematopoietic populations, trimming the networks of response genes whose expression was not reproduced.

One of the benefits of the integrated genomics approach is that it can implicate biological processes that would not have been detected using differential expression alone. The susceptibility networks that we identified are enriched in genes involved in DNA repair, base excision repair, apoptosis, and cell cycle, among other annotations. A second potential benefit of the integrated approach is that it differentiates between upstream (anchors) and response genes, an advantage over existing approaches that derive gene regulatory networks from expression data alone. While this is a hypothesis that remains to be tested, the identification of candidate upstream factors will be useful in prioritizing among apoptosis-related genes for experimental validation. For example, although *Casp9 *and *Bcl2 *are differentially expressed, *Casp9 *is also the candidate anchor of module A_33, the module most strongly associated with susceptibility status. We speculate that perturbation of candidate anchors, such as *Casp9*, are more likely to be informative in elucidating susceptibility than response genes (i.e. *Bcl2*).

Network analysis allowed us to predict the function of uncharacterized genes. For example, A630001G21Rik is expressed primarily in primitive hematopoietic and B-cells [41], yet its function is undetermined. Our analysis places it as the anchor of module A_12, which is enriched in apoptosis-related genes, including *Bcl2*. Therefore, A630001G21Rik may play previously unknown role in regulation of *Bcl2 *expression and apoptosis activity. Similarly, Cytoskeleton-associated protein-like 2 (*Ckap2l*) is the anchor of the largest module, A_16, enriched in both cell cycle and DNA repair genes. Although *Ckap2l *is highly expressed in hematopoietic progenitors [41], its functions are unknown. Its closest ortholog, *Ckap2*, is highly expressed in mouse stem cell lines and has detectable expression in hematopoietic progenitors, bone marrow, osteoclasts, osteoblasts, and macrophages [41]. There is a growing body of literature suggesting that *Ckap2 *(also known as Tumor-associated microtubule-associated protein) is involved in cell cycle progression [42-44]. It is possible that *Ckap2l *contributes to cell cycle regulation in HSCs and progenitors, and that genetic disturbances of its expression impact t-AML susceptibility. Experiments that perturb expression of anchor genes such as *Casp9 *and *Ckap2l *to assess their impact on module expression and activity are the next logical steps in determining the role of candidate networks in susceptibility. If such experiments demonstrate a causal link between anchor genes and module expression, then moving forward to formally define their role in leukemia will be warranted.

A drawback to the anchored network approach, as currently implemented, is that it assumes there is only a single anchor per module. In cases where CNVRs disrupt regulatory elements, it is possible that a single genetic event impacts the expression of multiple neighboring genes. For example, in module A_37 (Figure 4C) we found 10 response genes within 7 Mb of a CNVR. This module warrants special attention because it includes poly (ADP-ribose) polymerase family member 2 (*Parp2*, the anchor) and apurinic/apyrimidinic endonuclease 1 (*Apex1*), both members of the base excision repair pathway [45,46]. Both genes have lower expression in susceptible strains, again suggesting that lowered overall DNA damage response promotes susceptibility.

A caveat to the current work is that maps of genetic variation in the mouse genome are incomplete, a knowledge gap that promises to be filled by more informative SNP arrays [47] and next-generation sequencing [48,49]. It is possible that un-captured genetic variants may be the ultimate cause of the observed co-expression networks. These variants may mediate their impact through mechanisms other than altering the expression of anchors. In the extreme case, all modules may not be controlled by anchor expression, but by undetected causes. Nevertheless, the modules themselves are still informative in that they describe sets of coordinately regulated genes that, collectively, are associated with both t-AML susceptibility and biologically plausible processes and pathways.

## Conclusions

To our knowledge, this is the first report of an integrated genomics approach to dissect the role of the pre-exposure transcriptional state in t-AML susceptibility. From a clinical perspective, t-AML is important because the response to treatment is poor and survival is short [3]. But because t-AML is a clinically-induced malignancy, it is by definition preventable. Therefore, a long-term goal in this field is to gain sufficient understanding of susceptibility factors in order to make worthwhile the personalization of chemotherapeutic regimens based on t-AML risk. The transcriptional networks and their candidate anchors described here are an important early step towards gaining such an understanding.

## Methods

### Construction of SNP-based haplotype map

Genomic coordinates of 1,333 CNVRs were mapped from mm8 to mm9 using *liftOver*. 31 CNVRs were unmapped and dropped from further analysis. To derive haplotype blocks, SNPs for the haplotype map construction were downloaded from the Broad Institute http://www.broadinstitute.org/mouse/hapmap/. Only SNPs from 48 non-wild-derived strains were used. SNPs that were contained within CNVRs, had minor allele frequencies < 5%, or were not genotyped in 25% or more of strains were considered uninformative and were excluded from further analysis. The following steps were performed to simultaneously group SNPs into blocks and to assign haplotype to strains:

(1) Begin with the first informative SNP on a chromosome.

(2) If the number of SNPs in the current block is 1 then go to (3). Otherwise, go to (4).

(3) Group strains by genotype and add the next consecutive SNP to the current block.

(4) Cluster strains by SNP-based distance using *Partitioning Around Medioids*[50] (number of clusters = 2 to 6).

(5) Assign haplotype labels to strains based on the clustering with the maximum average silhouette [50].

(6) Derive consensus haplotypes. For each haplotype cluster, a consensus haplotype is defined as the string comprised of the most frequent genotype at each SNP position.

(7) Compare the consensus haplotypes to the actual SNP genotypes.

(8) If the number of errors is greater than 1 then go to (9), otherwise go to (10).

(9) Remove the most recently added SNP from the current block. Store the haplotyping results from the previous iteration. Start a new block with the current SNP. Go to (3).

(10) Add the next consecutive SNP to the current block. Go to (4). If there are no more SNPs on the current chromosome, select a new chromosome and go to (2). The computation is complete when all chromosomes have been analyzed.

SNP-based distances between strains are computed as the sum of SNP differences between strains. The range of number of allowable haplotypes per block was selected based on the estimated number of ancestral haplotypes [51]. Pooled multi-allelic R^2 ^was computed based on haplotype frequencies [52].

### Integrated expression QTL mapping

#### Pre-processing

GEP expression profiling was previously described [24] and is available at GEO under accession GSE10656. This data is referred to as kit+/lineage- (KL) throughout the text. Hypothalamus and adipose tissue expression data were obtained from GEO (accessions GSE5961 and GSE8028, respectively). For clustering and network analysis, probes were first filtered based on detection. In the KL data, a probe was considered detected in a sample if its signal was greater than a set of negative controls on the Illumina array. 13,496 probes were detected in all biological replicates of at least three strains (excluding C3H, for which only one array was analyzed). Only the 14,871 and 10,040 probes that were detected as present in at least 25% of the strains in the hypothalamus and adipose data sets, respectively, were kept for clustering analysis.

#### Cluster analysis

Unsupervised hierarchical clustering was performed with R's *hclust *function, using 1-Pearson correlation as the distance metric and the complete linkage method for node merging. To assess the non-randomness of the strains clustering according to susceptibility status, we computed the ratio of the mean of the distances among susceptible strains to the mean of the distances between all susceptible and resistant strains. Then, we permuted the strain labels 10,000 times, and recomputed the ratio of distances. The P-value of the observed clustering is the number of random permutations in which the distance test statistic > = observed distance test statistic divided by 10,000. This analysis was performed on the median expression profiles of strain replicates, only in those strains in which the susceptibility status is known. SNP clustering was based on strain-strain pair-wise distances computed by counting the number of SNPs that differ between each of the strains divided by the total number of SNPs that are typed in both strains.

#### Differential expression analysis

Strains with unknown susceptibility status were not included in the differential expression analysis. We used the *limma *package in R to model the expression of each gene with coefficients representing strain replicates and susceptibility status [53,54] and the false discovery rate (FDR) was estimated using q-value [55]. All of the 976 significant probes were detected as present in at least 50% of either the susceptible or resistant strains. When a gene is targeted by more than one probe, only the most significant differentially expressed probe was used for visualization. Association of module eigengenes with susceptibility was tested in the same way as differential expression. Enrichment analysis was performed using DAVID [56]. Only the GO annotations Biological Process 5 and KEGG pathways were assessed. We only report annotations that pass an FDR threshold < 25%. Expression data from all 20 strains previously profiled were used in expression network analysis. Anchored expression networks were identified by searching for probes that exhibited expression profiles that were significantly correlated with anchor gene expression at an FDR threshold < 1%.

#### Expression quantitative trait locus mapping

CNVR eQTLs previously identified were used in this analysis [24] and eQTLs based on SNP haplotypes were identified using the haplotype association method with weighted strain permutation to account for strain relatedness [57-59]

#### Analysis of coexpression networks

Normalized gene expression data used for validation of eQTLs and anchored modules was downloaded from GEO (GSE18067). This data set includes profiling on sorted (purified) hematopoietic stem, progenitor, myeloid and erythroid populations from female BXD recombinant inbred mice [29]. Only detection calls, coded as 0 for absent or 1 for present, were used to globally compare our KL data to the BXD data. Clustering was performed using the same parameters as described above for the KL data. KL eQTLs were validated by testing the association between the genotypes of SNPs within 2 Mb of anchor genes and driver gene expression in each compartment separately. Genotypes were treated as factors in a linear model of driver gene expression. P-values of the resulting F-statistics were adjusted for multiple testing using Holm's method [60]. Drivers that had corrected P-values < 0.05 in at least one compartment were considered validated. Assessing the reproducibility of the association between driver and response gene expression was performed in a similar manner. A linear model of response gene expression was fit with driver gene expression as the dependent variable (one model per driver-response gene pair per compartment). In this case, Benjamini and Hochberg's method to control the false discovery rate was applied to the resulting p-values [61]. WGCNA analysis was performed as previously described using the R package *WGCNA *[31]. Briefly, β values for calculating the weighted network adjacency were selected based on the power at which the scale law R^2 ^exceeded 0.9. Weighted adjacency matrices were computed, modules were defined using the *cut Tree Dynamic *function (which selects good dendrogram cutoffs) and similar modules were merged using *merge Close Modules *(which compensates for the high sensitivity of WGCNA). Eigengenes were computed as the first principal component of a module's expression matrix. Eigengenes were tested for differential expression between susceptible and resistant strains, as described above for individual genes.

## List of Abbreviations

(t-AML): therapy-related acute myeloid leukemia; (CNV): DNA copy number variation; (eQTL): expression quantitative trait loci; (SNPs): single nucleotide polymorphisms; (ENU): *N*-nitroso-*N*-ethylurea; (CNVRs): copy number variant regions; (GEP): gene expression profiling; (KL): c-kit+/lineage-; (FDR): false discovery rate; (WGCNA): Weighted Gene Co-expression Network Analysis; (*Casp9*): Caspase 9; (*Bcl-2*): B-cell leukemia/lymphoma 2; (*Bad*): BCL2-associated agonist of cell death; (*Bax*): BCL2-associated X protein; (*Msh6*): mutS homolog 6; (*O*6MeG): *O*6-methylguanine; (*Ckap2l*): Cytoskeleton-associated protein-like 2; (*Parp2*): poly (ADP-ribose) polymerase family member 2; (*Apex1*): apurinic/apyrimidinic endonuclease 1.

## Authors' contributions

PC and TG designed the study, performed analysis, and wrote the manuscript. Both authors read and approved the final draft.

## Supplementary Material

Additional file 1 **Supplementary Table S1**. SNP-derived haplotype blocks in 48 inbred mouse strains. Click here for file

Additional file 2 **Dendrograms showing clustering of strains by gene expression profile or SNP-derived haplotype blocks**. (A) Unsupervised clustering of strains using the strain median expression profile in KL cells groups strains by t-AML susceptibility status to an extent greater than expected by chance (see text), and differently than when clustering gene expression profiles of the hypothalamus (B), adipose tissue (C), or when clustering based on SNP-based distance (D). Click here for file

Additional file 3 **Heatmap of genes differentially expressed in KL cells from t-AML susceptible vs. resistant strains of mice**. 917 genes (976 probes) are differentially expressed between t-AML susceptible (SWR/J, DBA2/J, PL/J, AKR/J, BALB/cByJ) and t-AML resistant (C58/J, C57/J, FVB/J, C57BL/6J, A/J, NZB/J, C3H/HeJ, SJL/J, and 129S1/SvImJ) mice. Click here for file

Additional file 4 **Supplementary Table S2**. Annotation of genes differentially expressed between t-AML susceptible and resistant strains of mice. Click here for file

Additional file 5 **Unsupervised clustering of expression profiles of purified hematopoietic compartments**. BXD populations are indicated by the enriched population: erythrocytes (Ter119+, orange), myeloid lineage (Gr1+,green), hematopoietic stem cells (Lineage-Kit+Sca1+, red), and progenitors (Lineage-Kit+Sca1-, blue). Hematopoietic stem and progenitors from classical inbred strains are indicated by inbred strain name (Lineage-cKit+, navy blue). Each population forms a distinct cluster, with KL cells grouping most closely with stem and progenitor cells. Click here for file
