# Supplementary figures and images for: Integrated genomics of susceptibility to alkylator-induced leukemia in mice
Source: BMC Genomics. 2010 Nov 17;11:638. doi: 10.1186/1471-2164-11-638 (PMC3018144; doi:10.1186/1471-2164-11-638)

**A**

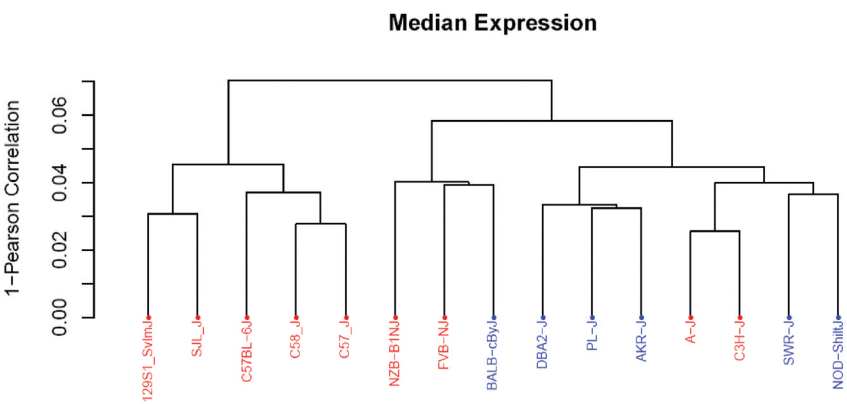

**B**

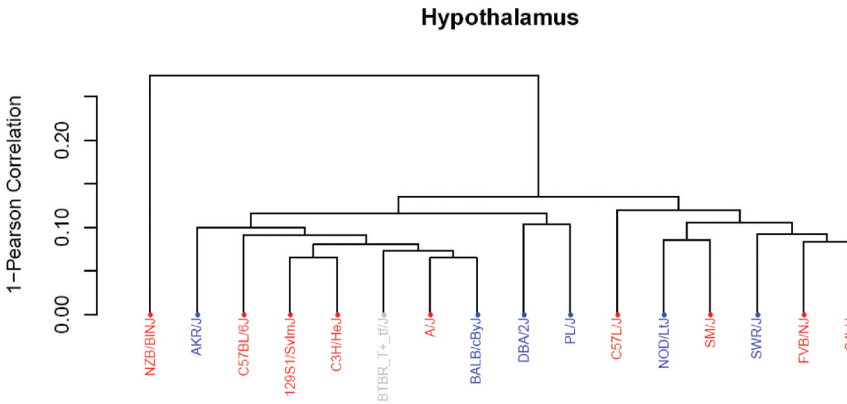

**C**

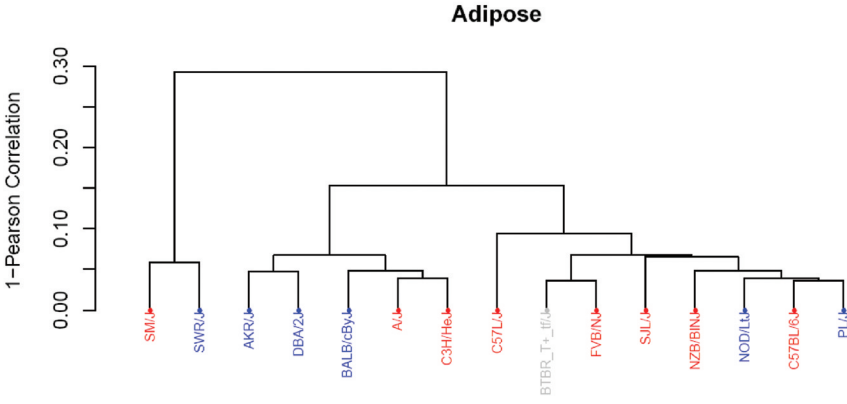

**D**

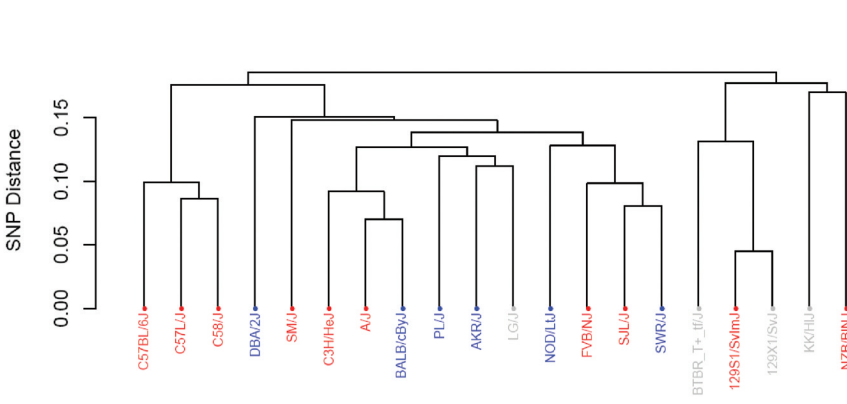

Supplement: Additional file 2 — Dendrograms showing clustering of strains by gene expression profile or SNP-derived haplotype blocks. (A) Unsupervised clustering of strains using the strain median expression profile in KL cells groups strains by t-AML susceptibility status to an extent greater than expected by chance (see text), and differently than when clustering gene expression profiles of the hypothalamus (B), adipose tissue (C), or when clustering based on SNP-based distance (D). [file 1471-2164-11-638-S2.PDF]

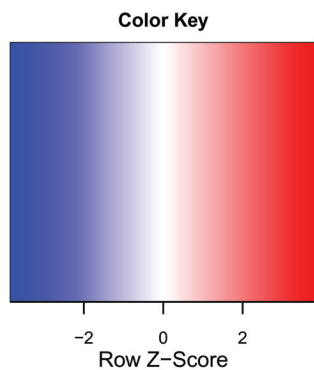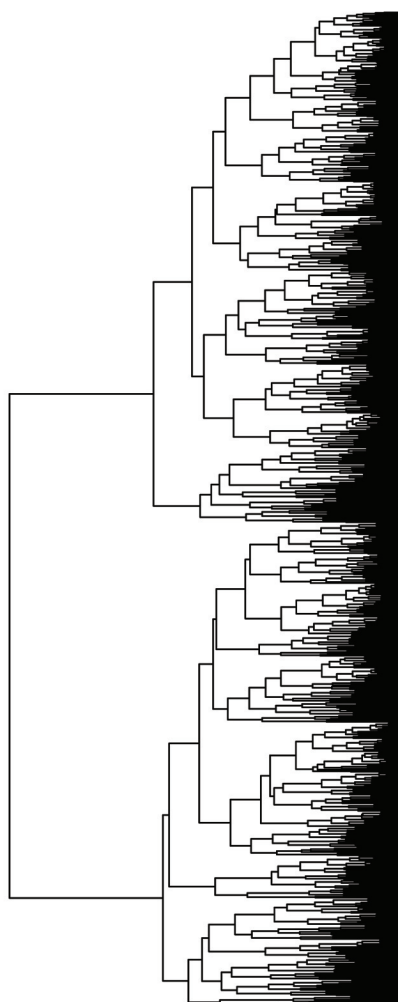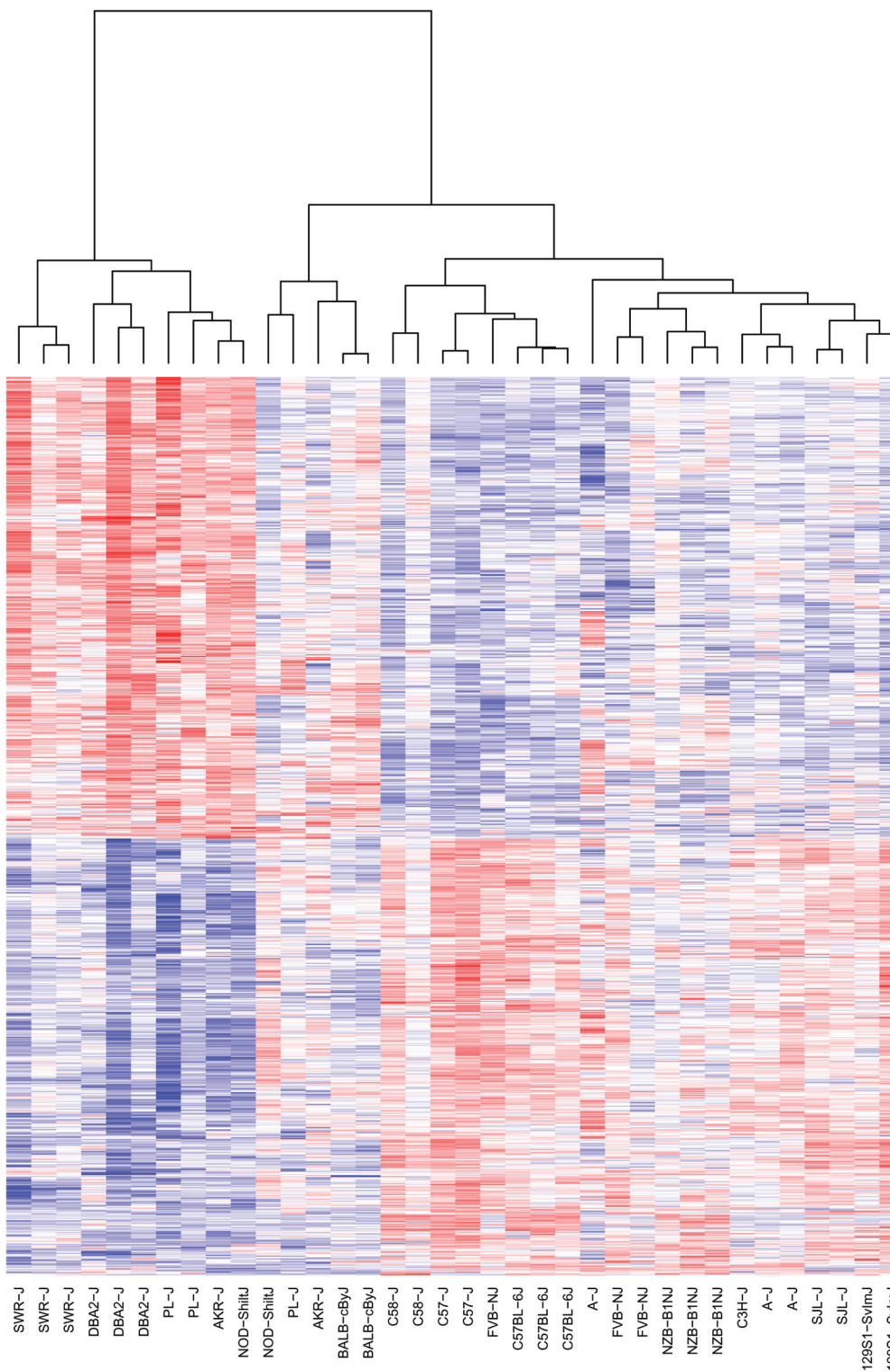

Supplement: Additional file 3 — Heatmap of genes differentially expressed in KL cells from t-AML susceptible vs. resistant strains of mice. 917 genes (976 probes) are differentially expressed between t-AML susceptible (SWR/J, DBA2/J, PL/J, AKR/J, BALB/cByJ) and t-AML resistant (C58/J, C57/J, FVB/J, C57BL/6J, A/J, NZB/J, C3H/HeJ, SJL/J, and 129S1/SvImJ) mice. [file 1471-2164-11-638-S3.PDF]

1-Pearson Correlation

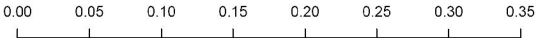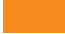

BXD Ter

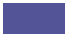

BXD Gr

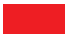

BXD KLS

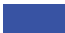

BXD KLS-

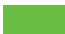

KL

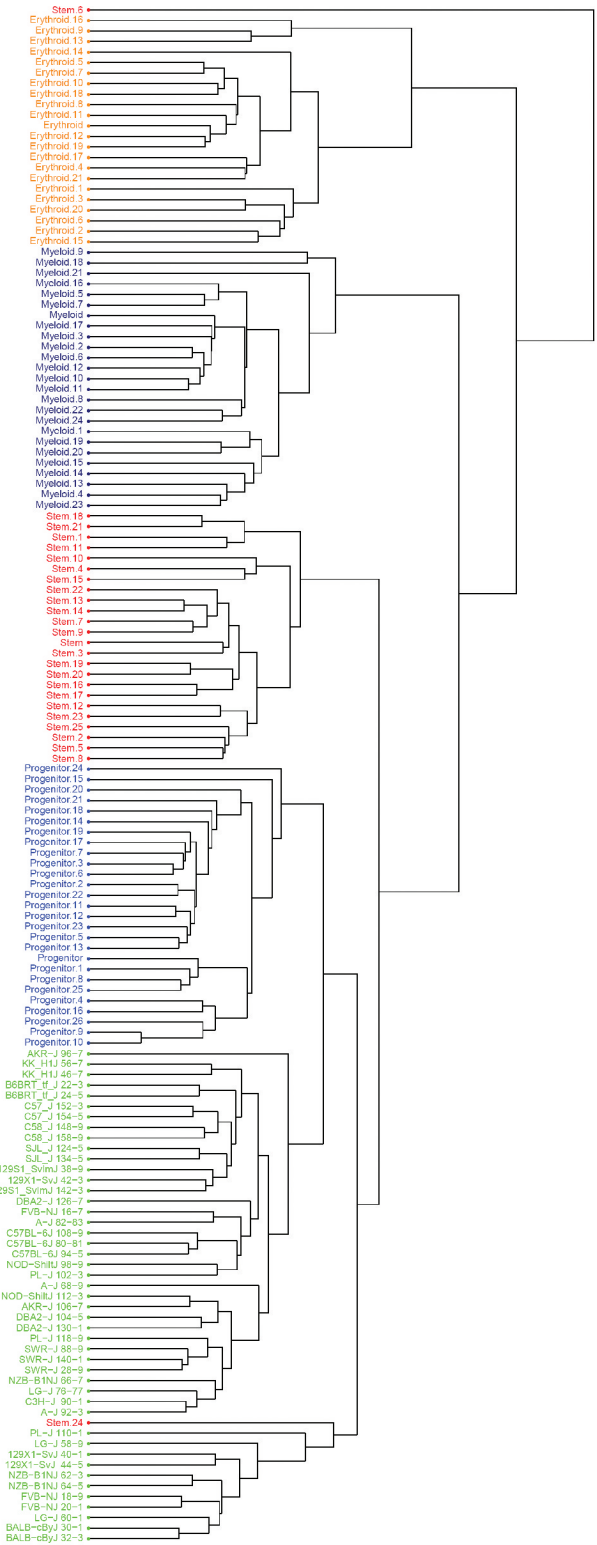

Supplement: Additional file 5 — Unsupervised clustering of expression profiles of purified hematopoietic compartments. BXD populations are indicated by the enriched population: erythrocytes (Ter119+, orange), myeloid lineage (Gr1+,green), hematopoietic stem cells (Lineage-Kit+Sca1+, red), and progenitors (Lineage-Kit+Sca1-, blue). Hematopoietic stem and progenitors from classical inbred strains are indicated by inbred strain name (Lineage-cKit+, navy blue). Each population forms a distinct cluster, with KL cells grouping most closely with stem and progenitor cells. [file 1471-2164-11-638-S5.PDF]
